# Effect of pressure controlled volume guaranteed ventilation during pulmonary resection in children

**DOI:** 10.1038/s41598-022-05693-y

**Published:** 2022-02-10

**Authors:** Change Zhu, Rufang Zhang, Shenghua Yu, Yuting Zhang, Rong Wei

**Affiliations:** 1 Department of Anesthesiology, Shanghai Children’s Hospital, Shanghai, China; 2 Department of Cardiothoracic Surgery, Shanghai Children’s Hospital, Shanghai, China

**Keywords:** Medical research, Outcomes research, Paediatric research

## Abstract

The purpose of the study was to evaluate the effect of pressure controlled volume guaranteed ventilation in children requiring one lung ventilation during pulmonary resection. Patients were randomly assigned to the lung protective ventilation combined with pressure controlled volume guaranteed group (PCV-VG group) or the lung protective ventilation combined with volume controlled ventilation group (VCV group). Both groups received tidal-volume ventilation of 8 ml kg^−1^ body weight during two lung ventilation and 6 ml kg^−1^ during OLV, with sustained 5 cmH_2_O positive end-expiratory pressure. Data collections were mainly performed at 10 min after induction of anaesthesia during TLV (T1), 5 min after OLV initiation (T2) and 5 min after complete CO2 insufflations (T3). In total, 63 patients were randomly assigned to the VCV (n = 31) and PCV-VG (n = 32) groups. The PCV-VG group exhibited lower PIP than the VCV group at T1 (16.8 ± 2.3 vs. 18.7 ± 2.7 cmH_2_O, *P* = 0.001), T2 (20.2 ± 2.7 vs. 22.4 ± 3.3 cmH_2_O, *P* = 0.001), and T3 (23.8 ± 3.2 vs. 26.36 ± 3.7 cmH_2_O, *P* = 0.01). Static compliance was higher in the PCV-VG group at T1, T2, and T3 (*P* = 0.01). After anaesthesia induction, lung aeration deteriorated, but with no immediate postoperative difference in both groups. Postoperative lung aeration improved and returned to normal from 2.5 h postextubation in both groups. PH was lower and PaCO_2_ was higher in VCV group than PCV-VG group during one lung ventilation. No differences were observed in PaO_2_-FiO_2_-ratio at T2 and T3, the incidence of postoperative pulmonary complications, intraoperative desaturation and the length of hospital stay. In paediatric patients, who underwent pulmonary resection requiring one lung ventilation, PCV-VG was superior to VCV in its ability to provide lower PIP, higher static compliance and lower PaCO_2_ at one lung ventilation during pneumothorax. However, its beneficial effects on different pathological situations in pediatric patients need more investigation.

## Introduction

One lung ventilation (OLV) has been widely used in children; however, it is associated with increased postoperative pulmonary complications according to adult research^1^. OLV is recognized as a risk factor for acute lung injury (ALI)^2^. ALI and acute respiratory distress syndrome are the leading causes of death after thoracic surgery, and they significantly reduce 1-year survival rate^3^. Paediatric patients have smaller functional residual capacities and larger closing volumes, rendering them having high airway pressure, low lung compliance, especially during one lung ventilation^4^. Children are vulnerable to ventilator induced barotrauma owing to high airway pressures during OLV^5^, which brings great concern to pediatric anesthesiologist^4^. Pressure controlled volume guaranteed ventilation (PCV-VG) is a novel type of ventilation mode. The decreasing airflow of PCV-VG allows airway pressure to achieve its maximum value at the beginning of inhalation and sustains the entire inhalation phase. Continuous plateau pressure in PCV-VG is more conducive for oxygen diffusion^6^. To date, several studies involving adults have demonstrated that PCV-VG potentially reduces airway pressure and improves lung compliance compared to volume controlled ventilation (VCV)^6–8^; however, the anatomical and physiological characteristics of children differ from adults. Whether children benefit from PCV-VG is unclear and relevant studies are lacking. On this premise, this study aimed to compare PCV-VG with VCV in terms of airway pressure, static compliance, PaO_2_-FiO_2_-ratio, PaCO_2_, arterial pH, lung aeration in lung ultrasound, postoperative pulmonary complications, intraoperative desaturation, hospital stay, and haemodynamics in children requiring OLV.

## Methods

**Figure 1:**
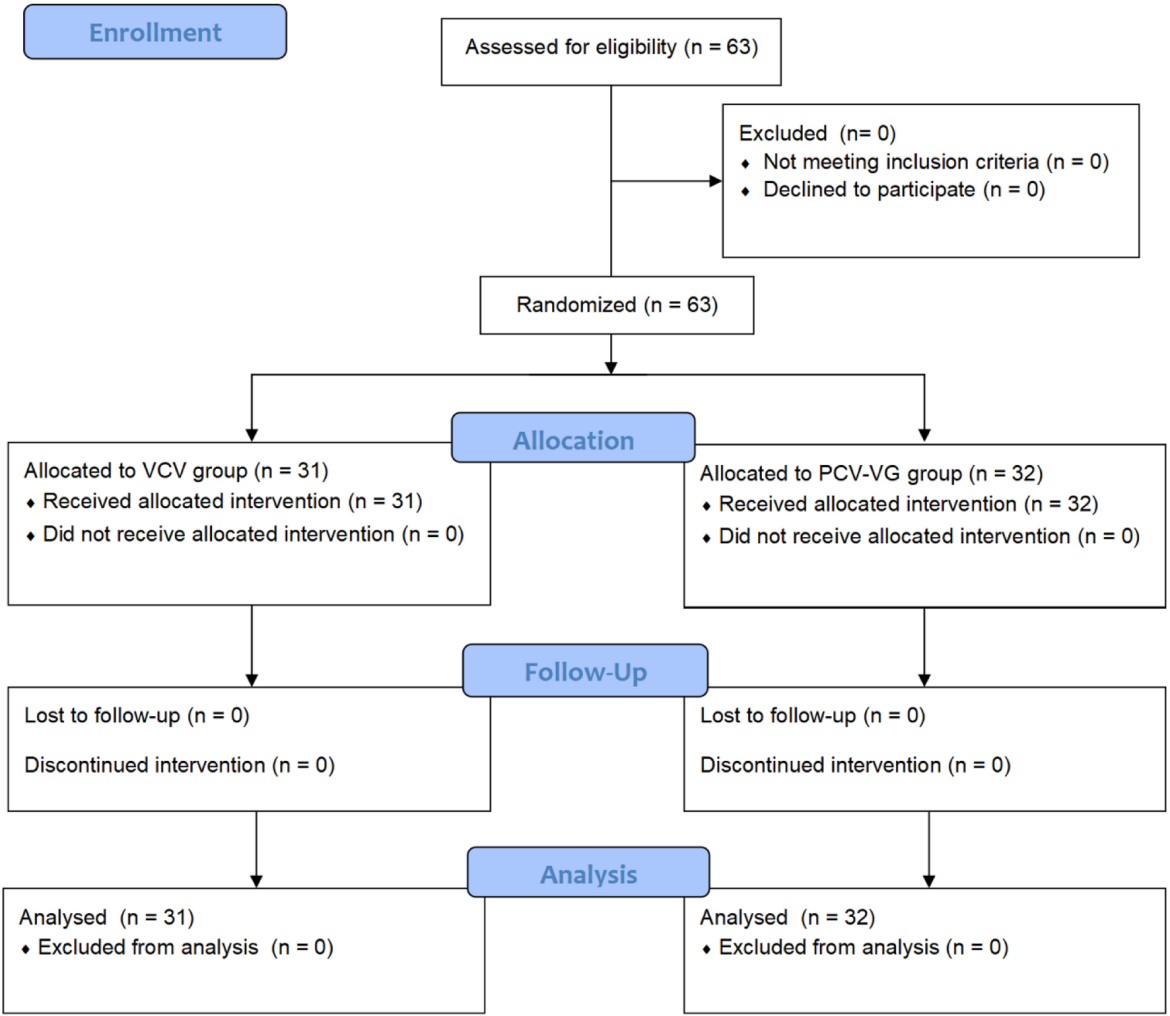
CONSORT flow diagram for patients included in the study.

The study was conducted in accordance with the principles of the Declaration of Helsinki^9^ after receiving approval from the Ethics Committee of Shanghai Children’s Hospital, Shanghai, China on 24 July 2019 (approval number: 2019R044-F01). The trial was registered in the Chinese Clinical Trial Registry at www.chictr.org.cn (trial number: ChiCTR2000035189, 02/08/2020). Informed consent was obtained from the parents or legal guardians of the children. This single-centre, prospective, randomised, controlled trial was conducted at a tertiary teaching children’s hospital affiliated with Shanghai Jiao Tong University, China, from 7 August 2020. The enrolment and allocation of patients are summarised in a CONSORT flow diagram (Fig. 1).

Inclusion criteria were as follows: Healthy paediatric patients aged 0–5 years with American Society of Anesthesiologists physical status 1 or 2 who required OLV while undergoing thoracoscopic surgery because of congenital cystic adenomatoid malformation. The exclusion criteria severe heart disease, other lung disease, upper respiratory tract infections, difficult airway or tracheotomy.

Computer-generated, sealed-envelope randomisation was performed to assign patients to one of the following two parallel arms in a 1:1 ratio, receiving different mechanical ventilation protocols: lung protective ventilation (LPV) combined with PCV-VG (PCV-VG group) or LPV combined with VCV (VCV group). One investigator (SZ) opened the envelopes and performed different mechanical ventilation protocols. The investigator did not participate in any other aspects of the trial. The patients as well as the Data Safety and Monitoring Board (DSMB) were also blinded to the random allocation.

All patients received a standardised general anaesthetic protocol, which included pre-oxygenation (without continuous positive airway pressure) and intravenous fentanyl (2 µg kg^−1^), propofol (3 mg kg^−1^), and rocuronium (0.6 mg kg^−1^). 5-Fr bronchial blocker (BB) was placed outside the endotracheal tube and placed into the target bronchi using fibre-optic bronchoscope. The pressure of the tracheal intubation cuff was maintained at 20–30 cmH_2_O^10^. All patients received ventilation using the same type of mechanical ventilator (Datex-Ohmeda-Avance CS2 Anesthesia Machine, GE Healthcare, Madison WI USA). At the end of one lung ventilation, the lungs were re-expanded manually with sustained inflation with 20–30 cmH_2_O of positive airway pressure for 10–15 s under direct observation to restore two lung ventilation^4,11^.

Anaesthesia was maintained using propofol 5–8 mg kg^−1^ h^−1^ to maintain the BIS (Philips Healthcare, Andover, MA) at 40–60 and remifentanil 0.1–0.4 µg kg^−1^ min^−1^ to maintain haemodynamic stability. Crystalloid solutions (6–8 ml kg^−1^ h^−1^) were used as maintenance fluids intraoperatively.

Ventilation settings in both groups were as follows:

Mechanical ventilation started with a tidal volume of 8 ml kg^−1^with PEEP at 5 cmH_2_O in both groups during two lung ventilation (TLV) and 6 ml kg^−1^with PEEP at 5 cmH_2_O in both groups during one lung ventilation. Volume controlled mode was used for mechanical ventilation in the VCV group and pressure controlled volume guaranteed ventilation mode was used in the PCV-VG group. The tidal volume was set based on the actual body weight. The inspired oxygen fraction was 0.5 during TLV and 1 during OLV. To ensure the end-expiratory concentration at 4.7–5.3 kPa, respiratory rate was adjusted at 18–25 breaths/min during TLV with an inspiratory/expiratory (I/E) ratio of 1:2. Respiratory rate was adjusted at 25–30 breaths min^−1^ during OLV with an I/E ratio of 1:2 to maintain an ETCO2 of no more than 7.9 kPa.

Measurements:

Data collections were performed during the following time points:

- Before induction of anaesthesia (T0).
- 10 min after induction of anaesthesia during TLV (T1).
- 5 min after OLV initiation (T2).
- 5 min after complete CO_2_ insufflations (T3).
- After wound closure before extubation (T4).
- 2.5 h after surgery (T5).

The following data were collected or calculated:

- Peak inspiratory pressure (PIP), static compliance.
- PaO_2_–FiO_2_-ratio, PaCO_2_ and arterial pH.
- Intraoperative desaturation (peripheral oxygen saturation (SpO_2_) < 90%).
- Postoperative pulmonary complications.

According to the recommendation of European joint taskforce published guidelines for perioperative clinical outcome (EPCO), the pulmonary complications were defined as respiratory infection, respiratory failure, pleural effusion, atelectasis, pneumothorax, bronchospasm, and aspiration pneumonitis^12,13^.

- Lung ultrasonography (LUS) in the dependent lung.

As reported in our previous study, LUS is an accurate method for diagnosing anesthesia-induced atelectasis in children. For the diagnosis of atelectasis, the sensitivity and accuracy of LUS was 88% and the specificity was 89% compared with MRI^14,15^. LUS was performed at the following three specific intervals: immediately before induction of anaesthesia (T0), immediately after wound closure before extubation (T4), 2.5 h after surgery (T5).

The four levels of aeration in LUS examination were classified as follows: N = 0, B1 = 1, B2 = 2, and C = 3^4,16,17^.

### Statistical analysis

Data are expressed as n (%), mean ± SD, or median (IQR) depending on the distribution of the data. Comparison of continuous variables between the study groups was performed using Student’s t-test for normally distributed data or the Mann–Whitney *U*-test for non-normally distributed data (we used the Shapiro–Wilk test to assess normality), and the χ^2^ test, Fisher’s exact test, as appropriate, for categorical variables. Comparison of the different variables over the study time points between the groups and within-group comparisons between the different time points was performed using repeated measures analysis of variances for normally distributed data. Statistical significance was set at P < 0.05.

The primary outcome was peak inspiratory pressure during OLV. A power analysis suggested that a minimum sample size of 26 patients for each group would be required to achieve a significance level of 5% with a power of 80%. The power was calculated from our preliminary data using an independent t-test, and the difference in mean peak inspiratory pressure between both modes of ventilation was 3 cmH_2_O, with a standard deviation of 3.8 cmH_2_O during OLV. The dropout rate was 20%, and 63 patients were sufficient. All statistical calculations were performed using the computer program SPSS version 25 (Statistical Package for the Social Science; IBM, Armonk, NY).

## Results

Under the supervision of the DSMB, patient enrolment commenced on 7 August 2020. In total, 63 patients were randomly assigned to the VCV (*n* = 31) and PCV-VG (*n* = 32) groups (Fig. 1). The baseline characteristics did not differ between the groups (Table 1).

**Table 1:** Baseline characteristics.

Parameters	VCV group (*n* = 31)	PCV-VG group (*n* = 32)	*P*-value
Age (month)	6.4 [5–40.75]	6.8 [5.2–39.3]	0.76
Weight (kg)	8.3 [7.5–13.25]	9 [7.55–12.3]	0.71
Sex (male/female)	25/6	24/8	0.59
**Type of operation**			
Segmentectomy/wedge resection	27	25	0.35
Single lobectomy	3	4	0.72
Bilobectomy	1	3	0.30
OLV time (min)	87.5 [71–118.75]	106 [60.25–125]	0.44
Total operation time (min)	121.5 [101.25–153.75]	145 [93.25–162]	0.46
Anesthesia time (min)	167 [144.25–192.75]	182.0 [137.5–201.75]	0.66
Respiratory rate_T1_ (min^−1^)	20.4 ± 0.9	20.1 ± 0.4	0.14
Respiratory rate_T2_ (min^−1^)	26 [25–27]	26 [25–27]	0.27
Respiratory rate_T3_ (min^−1^)	30 [25–30]	25 [25–30]	0.26

All data are presented as mean ± SD or median [IQR], unless otherwise specified.

*VCV* volume controlled ventilation, *PCV-VG* pressure controlled volume guarantee ventilation, *OLV* one lung ventilation.

### Peak inspiratory pressure

**Figure 2:**
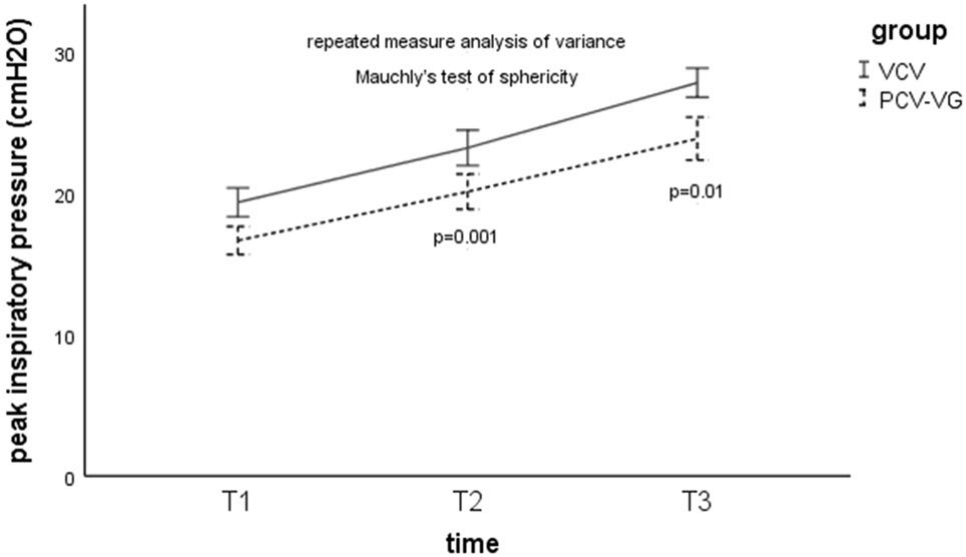
Peak inspiratory pressure in the two groups at different stages of the study. T1, 10 min after induction of anaesthesia in the supine position without pneumothorax; T2, 5 min after OLV commencement; T3, 5 min after complete CO_2_ insufflations; *VCV* volume controlled ventilation, *PCV-VG* pressure controlled volume guaranteed ventilation. The data was presented as mean (standard deviation).

For the comparison of PIP between the groups, the results of repeated measures ANOVA revealed PIP at T2 and T3 were lower in the PCV-VG group than in the VCV group (T2, 20.2 ± 2.7 cmH_2_O vs 22.4 ± 3.3 cmH_2_O; P = 0.001) (T3, 23.8 ± 3.2 cmH_2_O vs 26.36 ± 3.7 cmH_2_O; P = 0.01) (Fig. 2). For the comparison of PIP within groups, the Mauchly’s test of sphericity, P > 0.05, indicated that the dataset satisfied the sphericity assumption, and the results of repeated measures ANOVA revealed PIP was higher at T2 and T3 compared with T1 in both groups (*P* = 0.001) (Fig. 2).

### Static compliance

**Figure 3:**
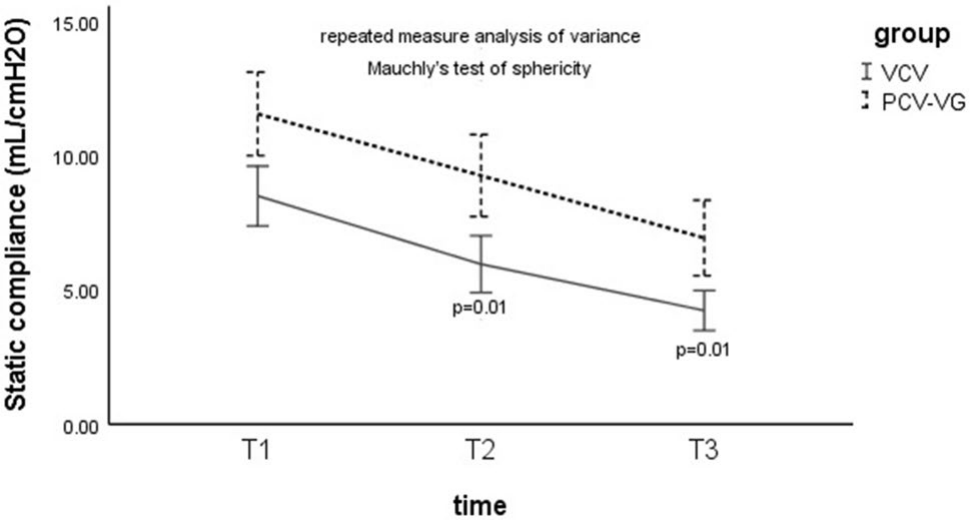
Static compliance in the two groups at different stages of the study. T1, 10 min after induction of anaesthesia in the supine position without pneumothorax; T2, 5 min after OLV commencement; T3, 5 min after complete CO_2_ insufflations; *VCV* volume controlled ventilation, *PCV-VG* pressure controlled volume guaranteed ventilation. The data was presented as mean (standard deviation).

For the comparison of static compliance between the groups, the results of repeated measures ANOVA revealed static compliance at T2 and T3 was higher in the PCV-VG group than in the VCV group (T2, 9.1 ± 3.7 ml/cmH_2_O vs 6.8 ± 3.05 ml/cmH_2_O; P = 0.01) (T3, 7.1 ± 3.3 ml/cmH_2_O vs 4.8 ± 2.3 ml/cmH_2_O; P = 0.01) (Fig. 3).

For the comparison of static compliance within groups, the Mauchly’s test of sphericity, P = 0.017, indicated that the dataset violated the sphericity assumption, therefore we accepted the result of the multivariate tests, Pillai’s trace, P = 0.0001, indicating static compliance was lower at T2 and T3 compared with T1 in both groups (*P* = 0.01) (Fig. 3).

### LUS assessment

**Figure 4:**
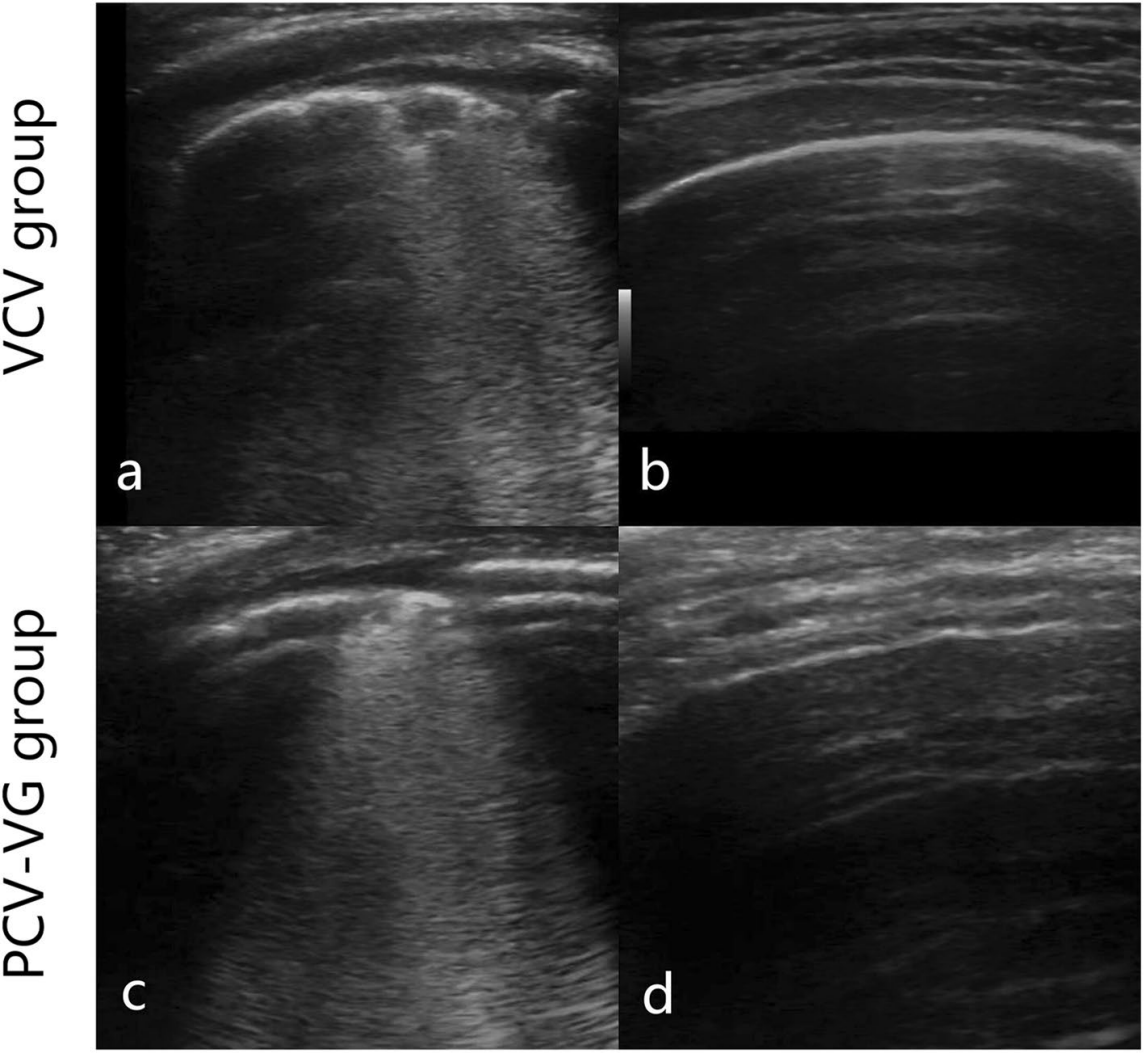
Lung ultrasound findings of dependent lung in the PCV-VG and VCV groups. The images were obtained from the posterior region of the dependent lung. (**a**) Lung aeration after surgery in VCV group. (**c**) Lung aeration after surgery in PCV-VG group. (**b**) Lung aeration 2.5 h after surgery in VCV group. (**d**) Lung aeration 2.5 h after surgery in PCV-VG group. *VCV* volume controlled ventilation, *PCV-VG* pressure controlled volume guaranteed ventilation.

There was no difference in LUS in the dependent lung before T0. After T0, lung aeration deteriorated, but with no difference in both groups immediately after wound closure before extubation [T4: 4 (2 to 6) vs. 4 (2 to 5) Z = – 0.69, *P* = 0.49] (Fig. 4a,c). Lung aeration improved in both groups after surgery and returned to normal from 2.5 h after extubation in both groups (Fig. 4b,d). Temporal ultrasound images of the lateral chest wall of the dependent lung are shown in Fig. 4.

### Postoperative pulmonary complications

Five (7.9%) patients exhibited postoperative pulmonary complications, with no differences in the incidence between the two groups [VCV: 3 (9.7%) vs. PCV-VG: 2 (6.3%), *P* = 0.97].

### PaO_2_–FiO_2_-ratio, PaCO_2_, arterial pH and intraoperative desaturation

The data of PaO_2_–FiO_2_-ratio at T2 was non-normally distributed data according to Shapiro–Wilk test. Comparison of PaO_2_–FiO_2_-ratio was performed using the Mann–Whitney U-test, and there was no difference in PaO_2_–FiO_2_-ratio at T2 between VCV group [54 (32.2 to 62.6 kPa)] and PCV-VG group [55.9 (50.4 to 63.3 kPa)] (z = − 0.858; *P* = 0.39).

The data of PaO_2_–FiO_2_-ratio, pH and PaCO_2_ at T3 were normally distributed data according to Shapiro–Wilk test. Comparisons between the groups were performed using the Student’s t-test, and there was no difference in PaO_2_–FiO_2_-ratio at T3 between VCV group (34.6 ± 7.1 kPa) and PCV-VG group (37.9 ± 8.0 kPa) (*P* = 0.35). However, pH was lower in VCV group (7.19 ± 0.06) than PCV-VG group (7.28 ± 0.05), (*P* = 0.008). PaCO2 was higher in VCV group (59.9 ± 6.4 mmHg) than PCV-VG group (51.3 ± 7.2 mmHg), (*P* = 0.013).

The incidence of intraoperative desaturation was comparable in VCV group (4/31, 12.9%), compared with 3/32 (9.4%) in the PCV-VG group [OR = 1.43 (0.29 to 7.0); *P* = 0.66].

### The length of hospital stays

The length of hospital stay did not differ between the PCV-VG (6.5 ± 2.1 days) and VCV (6.1 ± 1.9 days) groups. (*P* = 0.43).

### Haemodynamic variables

**Figure 5:**
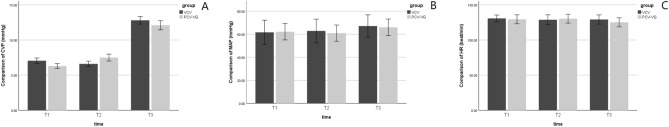
Comparison of haemodynamic variables at different time points. (**A**) CVP, central venous pressure; (**B**) MAP, mean arterial pressure; (**C**) HR, heart rate VCV, volume controlled ventilation; PCV-VG, pressure controlled volume guaranteed ventilation; T1, 10 min after induction of anaesthesia without pneumothorax; T2, 5 min after OLV commencement; T3, 5 min after complete CO_2_ insufflation.

There was no difference in the haemodynamic variables between the groups. MAP was higher at T3 in both groups compared with that at T1 and T2 (*P* = 0.001). CVP was higher at T2 and T3 than that at T1 (*P* = 0.001). The heart rate was stable throughout the operation (Fig. 5).

## Discussion

This randomised controlled trial revealed that PCV-VG was superior to VCV in its ability to provide ventilation with lower PIP, lower PaCO2 and higher static compliance and PH during one lung ventilation. After anaesthesia induction, lung aeration deteriorated, but with no immediate postoperative difference in both groups. Postoperative lung aeration improved and returned to normal from 2.5 h postextubation in both groups. No differences were observed in PaO_2_–FiO_2_-ratio at T2 and T3, the incidence of postoperative pulmonary complications and intraoperative desaturation and the length of hospital stay. However, its beneficial effects on different pathological situations in pediatric patients need more investigation.

Similar results have been obtained in laparoscopic^6,7^ and lumbar spine surgeries^8^. High airway pressures achieved during OLV have reportedly been associated with postpneumonectomy pulmonary oedema^4,18^ and acute lung injury after pneumonectomies^19^. Therefore, patients undergoing OLV may benefit more from lower PIP. To the best of our knowledge, evidence-based recommendations for ventilation strategies during OLV in children are lacking and this is the first randomised controlled trial to evaluate the effect of LPV combined with PCV-VG during OLV in a paediatric population.

Patients who received PCV-VG exhibited lower PIP and higher static compliance, indicating a possible association with the decreasing airflow of PCV-VG, which allows airway pressure to achieve its maximum at the beginning of inhalation and continue the entire inspiratory phase^6^. Continuous measurement of lung compliance and volumetric pressure automatically adjusts the air supply flow rate and air pressure^7^. Therefore, PCV-VG mode potentially reduces airway pressure to the greatest extent possible while ensuring ideal volume and improving lung compliance^6–8,20^.

In our study, lung aeration was comparable in both groups after surgery and was fully restored 2.5 h after surgery, as evaluated using LUS. This result is consistent with that of our previous study^17^. However, the influence of the ventilation mode can easily overlap because patient factors or other factors affect patient prognosis to a greater extent^20^. For instance, the lungs were manually re-expanded at the time of thoracic cavity closure. Moreover, patient may exhibit compensatory adaptations, which need to be considered^20^. To be more specific, patient with healthy lungs may compensate and overcome perioperative lung problems, such as lung oedema or atelectasis, but this is unlikely in patients with ALI or acute respiratory distress syndrome.

In the current study, PaO_2_ increased significantly in both groups at T3 compared to that at baseline, which might be explained by inspired oxygen concentration of 0.5 with air during TLV and 1 during OLV. Additionally, no superiority in PaO_2_–FiO_2_-ratio was observed regardless of the mode of ventilation, a finding that corroborates with previous studies^6,21^. This finding may be explained by the similarity in the mean values^6^. Additionally, we found no difference in the postoperative pulmonary complications (PPCs) between the two groups. This may be related to limited fluid input, plateau pressures remaining below 30 cmH_2_O at all times^22^, and lung protective ventilation^4^. Most important, the sample was too small.

However, this study also had certain limitations. First, blinding was not conducted in investigators who were aware of the mode of ventilation. Second, we did not enroll patients with obesity or lung injuries. Therefore, its beneficial effects on different pathological situations in pediatric patients need more investigation.

In summary, PCV-VG mode reduced the airway pressure and PaCO2, increased static compliance and PH during one lung ventilation in children undergoing pulmonary resection.
